# Validation of improved 24-hour dietary recall using a portable camera among the Japanese population

**DOI:** 10.1186/s12937-021-00724-2

**Published:** 2021-07-15

**Authors:** Yumi Matsushita, Tosei Takahashi, Kumiko Asahi, Emiko Harashima, Hiroko Takahashi, Hiroyuki Tanaka, Yoshiko Tsumuraya, Nobuko Sarukura, Masashi Furuta, Heizo Tanaka, Tetsuji Yokoyama

**Affiliations:** 1 Department of Clinical Research, National Cen, ter for Global Health and Medicine, 1-21-1 Toyama, Shinjuku-ku, Tokyo, 162-8655 Japan; 2 Department of Nutritional and Health Sciences, Faculty of Food and Nutritional Sciences, Toyo University, 1-1-1 Izumino, Itakura-machi, Ora-gun, Gunma 374-0193 Japan; 3 Department of Clinical Nutrition, Faculty of Health and Wellness Sciences, Hiroshima International University, 5-1-1 Hirokoshinkai, , Kure city, Hiroshima 737-0112 Japan; 4 Department of Nutrition and Life Science, Faculty of Health and Medical, Sciences, Kanagawa Institute of Technology, 1030 Shimo-ogino, Atsugi-shi, Kanagawa, 243-0292 Japan; 5 The Japanese Society of Nutrition and Dietetics, 3-4-18-904 Mita Minato-ku, Tokyo, 108-0073 Japan; 6 Department of Human Nutrition Faculty of Human Nutrition, Tokyo Kasei Gakuin University, 22 Sanbant-cho Chiyoda-ku, Tokyo, 102-8341 Japan; 7 Department of Nutritional Management, Faculty of Nutritional Science, Sagami Women’s University, 2-1-1 Bunkyo, Minami-ku, Sagamihara-shi, Kanagawa, 252-0383 Japan; 8 Department of Nutrition and Health, Faculty of Nutritional Science, Sagami Women’s University, 2-1-1 Bunkyo, Minami-ku, Sagamihara-shi, Kanagawa, 252-0383 Japan; 9 Department of Nutrition, Toho University Omori Medical Center, 6-11-1 Omori-Nishi, Ota-ku, Tokyo, 143-8541 Japan; 10 Professor Emeritus, Tokyo Medical and Dental University, 1-5-45 Yushima Bunkyo-ku, Tokyo, 113-8510 Japan; 11 Department of Health Promotion, National Institute of Public Health, 2-3-6 Minami, Wako, Saitama 351-0197 Japan

**Keywords:** 24-h dietary recall, Weighed food records, Validation

## Abstract

**Background:**

The collection of weighed food records (WFR) is a gold standard for dietary assessment. We propose using the 24-h recall method combined with a portable camera and a food atlas (24hR-camera). This combination overcomes the disadvantages of the 24-h dietary recall method. Our study examined the validity of the 24hR-camera method against WFR by comparing the results.

**Methods:**

Study subjects were 30 Japanese males, aged 31–58 years, who rarely cook and reside in the Tokyo metropolitan area. For validation, we compared the estimated food intake (24hR-camera method) and weighed food intake (WFR method). The 24hR-camera method uses digital photographs of all food consumed during a day, taken by the subjects, and a 24-h recall questionnaire conducted by a registered dietitian, who estimates food intake by comparing the participant’s photographs with food atlas photographs. The WFR method involves a registered dietitian weighing each food item prepared for the subject to consume and any leftovers. Food intake was calculated for each food group and nutrient using the 24hR-camera vs. weighed methods.

**Results:**

Correlation coefficients between the estimated vs. weighed food intake were 0.7 or higher in most food groups but were low in food groups, such as oils, fats, condiments, and spices. The estimated intake of vegetables was significantly lower for the 24hR-camera method compared to the WFR method. For other food groups, the percentages of the mean difference between estimated vs. weighed food intake were -22.1% to 5.5%, with no significant differences between the methods (except for algae, which had a very low estimated intake). The correlation coefficients between the two methods were 0.774 for energy, and 0.855, 0.769, and 0.763 for the macronutrients, proteins, lipids, and carbohydrates, respectively, demonstrating high correlation coefficients: greater than 0.75. The correlation coefficients between the estimated vs. weighed for salt equivalents and potassium intake were 0.583 and 0.560, respectively, but no significant differences in intake were observed.

**Conclusions:**

The 24hR-camera method satisfactorily estimated the intake of energy and macronutrients (except salt equivalents and potassium) in Japanese males and was confirmed as a useful method for dietary assessment.

## Background

Collecting weighed food records (WFR) is a gold standard for accurate dietary assessment; however, it is not practical to weigh food and drink at every meal and calculate their nutritional content. The 24-h dietary recall method is widely used in general dietary assessment [1]. This method employs trained interviewers to collect of dietary intake data based on the participants’ memory. The major disadvantages of this method are that it is time-consuming, relies on the participant's memory, and is prone to omissions of food items. To overcome these disadvantages, we propose the 24-h recall method which uses a portable camera and a food atlas (24hR-camera) [2]. The food atlas details portion sizes for various foods commonly consumed in Japan. Our study examined the validity of the 24hR-camera method by conducting a simultaneous WFR study and comparing results.

## Methods

### Participants

The study subjects were recruited through Japanese universities in Tokyo, Gunma, Kanagawa, and Shizuoka prefectures. Fathers of students were recruited if they lived in the same household and rarely cooked. Thirty Japanese males, aged 31-58, residing in the Tokyo metropolitan area participated in the study. The criterion of “rarely cooks” was defined as “seldom cooks for themselves or others, and usually eat food prepared for them by someone else” and was deliberately chosen with the assumption that the participants had very little sense of food weight; resulting in inaccurate recognition of food intake.

This research was conducted after approval was obtained from the ethics committee of Kanagawa Institute of Technology (Kanagawa, Japan). Written informed consent was obtained voluntarily from all participants.

### Survey methodology

**Fig. 1:**
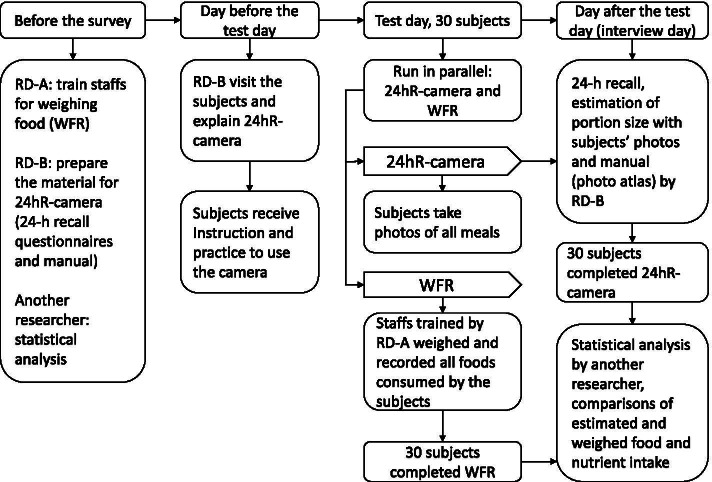
Design of the survey: Validation of the 24hR-camera by comparison against WFR

Figure 1 shows the design of the survey. Before data collection, registered dietitians (RD) were divided into three groups: RD-A administered WFR, RD-B administered 24hR-camera, and the third RD conducted the statistical analysis. RD-A trained staff to conduct weighing of food items. RD-B explained the use of the 24hR-camera to participants on the day before data collection. Participants were also asked not to eat takeaway or restaurant meals for the duration of the 24-h study period. WFR was conducted within one day, and 24hR-camera was divided into two consecutive days (test and interview day). On the test day, WFR and 24hR-camera were performed simultaneously.

### Weighed Food Record (WFR)

On the test day, food and drink were prepared at the participant’s home, in a separate room from participants. The pre-cooked ingredients, cooked meals and drinks were weighed before the food was served to participants by staff trained by the RD-A. Leftovers were weighed to calculate the actual intake amount.

### 24hR-camera method

**Fig. 2:**
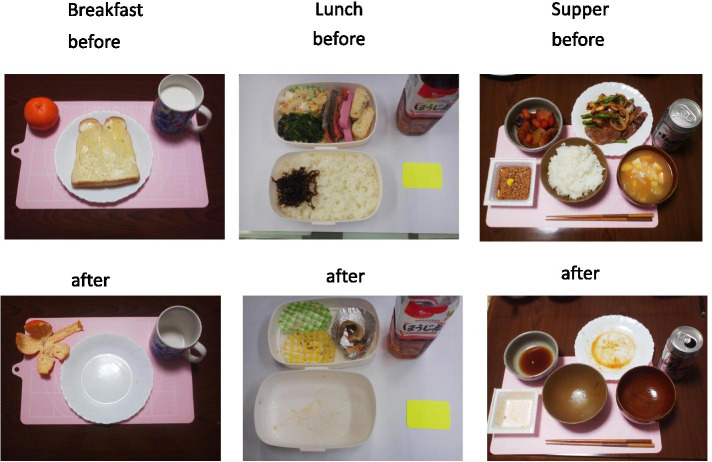
Examples of photographs of breakfast, lunch, and supper. The actual weight of foods consumed was calculated by comparing before and after each meal

The same meals, snacks and drinks were used in the WFR and 24hR-camera methods. On the simultaneous test day for the 24hR-camera method, participants photographed every food and drink item before and after consumption during a 24-h period (AM0 to PM12). Photographs of meals were taken with a card-sized sheet of colored paper placed by the food or a gridded mat under the meal. Photographs were taken at breakfast, lunch, supper, and between meals on the test day (examples are shown in Fig. 2).

The following day (interview day), RD-B interviewed each participant and recorded the estimate of food and drinks consumed, based on each participant's photographs and memory. RD-B contrasted participants’ photographs against a food atlas manual [2], which lists the variety of foods commonly consumed in Japan and includes printed photographs of food in full-scale portion size. RD-B compared the actual meal photographs with the manual photos and completed the record sheets. The less common food items, not listed in the food atlas, were replaced with similar food items in the atlas. The record sheets consisted of the meal time (including consumption between meals), location, dishes, foodstuffs, portion size, food preparation, amount of leftover food, and intake of any supplement. RD-B estimated food weight and dietary intake. The weight of food intake was estimated using the following procedure: 1) breakfast, lunch, supper, and between-meal snacks were clearly distinguished; 2) each dish was named; 3) food components making up the dish were extrapolated; 4) food components were classified into the components listed in the Standard Tables of Food Composition in Japan; 5) seasonings and condiments used were extrapolated; 6) the net intake weight of foods was estimated. Further details of the method are shown in the manual [2].

### Calculation of nutritional value

RD-A calculated the nutritional values for the WFR data, and RD-B calculated the nutritional values for the 24hR-camera data, using the *Standard Tables of Food Composition in Japan, Fifth Revised and Enlarged Edition 2010* [3].

### Body mass index

Body mass index (BMI) was calculated from the self-reported height and weight of each participant (BMI = weight [kg] / height [m^2^]).

### Statistical analysis

The ingested foods were divided into 17 food groups, based on the *Standard Tables of Food Composition* [3]. Food intake values (g) were summarized as mean and standard deviation (SD). The 24hR-camera method was validated by comparison with the WFR method, using mean difference and Spearman’s correlation coefficient. We used Spearman's correlation coefficients because the distributional distortions varied among food categories and nutrients.

The Bland–Altman plot, a graphical tool to compare the measurements of different methods, was used to examine the agreement between the 24hR-camera and WFR data [4]. The mean difference between estimated (24hR-camera) vs. weighed (WFR) intakes for each food group or nutrient was plotted against its mean value for each subject. Percentages of the mean difference between 24hR-camera and WFR in each food category were calculated as the percentage of the difference (mean amount from 24hR-camera minus mean amount from WFR)/ mean amount from WFR)*100. We determined any implausible intakes by examining each food item in the low or high intake range. All analyses were performed using SPSS for Windows version 27.0 (SPSS, Chicago, IL).

## Results

All 30 subjects completed both the WFR and the 24hR-camera methods. The characteristics of the subjects are shown in Table 1.

**Table 1:** Age and anthropometric values of the subject

		Mean	SD
Age	years	49.3	6.8
Height	cm	170.4	4.5
Weight	kg	70.4	11.6
BMI	kg/m^2^	24.2	3.5

*n* = 30

**Fig. 3:**
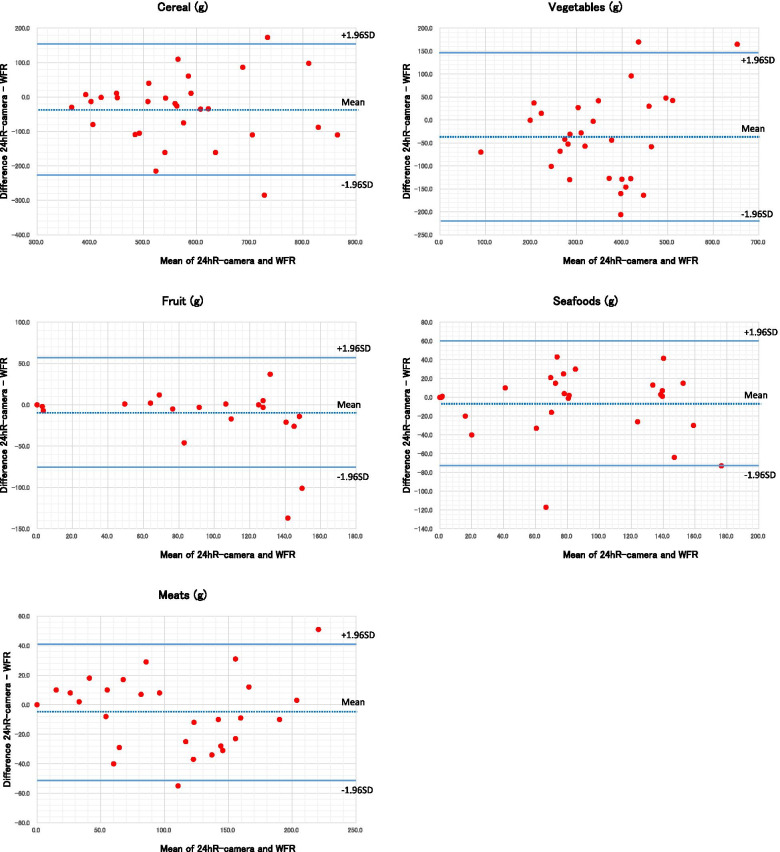
Comparison of food intake between the 24hR-camera and WFR methods, using Bland–Altman plots in 30 Japanese men. The differences between the amounts of foods estimated by 24hR-camera and WFR (Y-axis) were plotted against their mean values (X-axis). The dotted line shows the average difference between the two methods; the smaller the difference, the smaller the systematic bias. The solid lines show the 95% limits of agreement representing the range in which most differences are expected to fall; the smaller the range, the smaller the difference between two methods for most individuals

A comparison of the estimated (24hR-camera) vs. weighed (WFR) food intake for each food group is shown in Table 2 and Fig. 3. There were no implausible intakes, either in the low or high range. Correlation coefficients between the 24hR-camera vs.WFR intake were 0.7 or higher in most food groups but were low in food groups that are difficult to visually discern, such as oils, fats, condiments, and spices. The intake of vegetables was significantly lower in the estimated compared to the weighed. For other food groups (except algae, in which the intake amount was very low), the differences between the two groups were -22.1% to 5.5%, with no significant differences between the estimated and weighed intake.

**Table 2:** Mean difference and correlation coefficient of food intakes between 24hR-camera and WFR in 30 Japanese men

	24hR-camera	WFR	Difference: 24hR-camera - WFR	P^b^	Correlation coefficient	95% Limits of agreement^c^	
Food category (g)	Mean ± SD	Mean ± SD	Mean difference (%)^a^		Spearman (95%CI)		
01: Cereals	553.5 ± 139.5	589.5 ± 143.1	-6.1	0.053	0.783 (0.589, 0.892)	-227.4 (-39.8)	155.6 (27.2)
02: Potatoes and Starches	54.3 ± 63.5	69.7 ± 92.5	-22.1	0.160	0.897 (0.793, 0.950)	-130.2 (-210.1)	99.4 (160.3)
03: Sugars and Sweeteners	5.5 ± 7.1	4.6 ± 4.8	21.1	0.329	0.514 (0.189, 0.738)	-9.4 (-187.1)	11.3 (225.2)
04: Pulses	63.1 ± 62.8	66.7 ± 63.4	-5.4	0.637	0.764 (0.557, 0.882)	-84.7 (-130.5)	77.5 (119.4)
05: Nuts and Seeds	3.8 ± 12.1	3.9 ± 12.4	-2.2	0.891	0.719 (0.484, 0.857)	-6.6 (-171.9)	6.4 (167.5)
06: Vegetables	336.2 ± 132.1	372.0 ± 111.7	-9.6	0.045	0.738 (0.515, 0.868)	-218.8 (-61.8)	147.3 (41.6)
07: Fruits	77.6 ± 80.9	86.6 ± 83.4	-10.4	0.157	0.906 (0.810, 0.955)	-75.5 (-92.0)	57.5 (70.0)
08: Mushrooms	29.8 ± 44.6	31.2 ± 35.3	-4.2	0.805	0.889 (0.778, 0.946)	-58.0 (-190.2)	55.4 (181.6)
09: Algae	10.0 ± 12.8	6.4 ± 9.7	56.3	0.052	0.855 (0.715, 0.929)	-15.5 (-188.6)	22.7 (276.5)
10: Fishes and Shellfishes	75.0 ± 57.3	81.3 ± 60.4	-7.7	0.317	0.852 (0.710, 0.928)	-72.6 (-92.9)	60.0 (76.8)
11: Meats	96.6 ± 63.7	101.4 ± 64.5	-4.8	0.272	0.946 (0.889, 0.974)	-51.2 (-51.7)	41.5 (41.9)
12:Eggs	53.5 ± 44.7	50.7 ± 37.4	5.5	0.429	0.865 (0.733, 0.934)	-34.7 (-66.5)	40.3 (77.3)
13: Dairy Products	88.2 ± 101.7	101.6 ± 112.4	-13.2	0.053	0.970 (0.937, 0.986)	-84.4 (-88.9)	57.6 (60.7)
14: Fats and oils	14.5 ± 10.4	12.8 ± 9.0	13.1	0.356	0.533 (0.214, 0.749)	-17.5 (-128.7)	20.9 (153.4)
15: Confectioneries	18.3 ± 37.8	18.2 ± 38.4	0.6	0.871	0.969 (0.935, 0.985)	-6.5 (-35.5)	6.7 (36.6)
16: Beverages	673.6 ± 382.8	661.3 ± 357.4	1.9	0.399	0.955 (0.907, 0.979)	-142.1 (-21.3)	166.7 (25.0)
17: Seasonings and Spices	176.2 ± 135.3	153.3 ± 148.5	15.0	0.448	0.465 (0.126, 0.707)	-297.1 (-180.3)	342.9 (208.1)

*SD* standard deviation, *CI* confidence interval

^a^Persentage of mean difference between 24hR-camera and WFR in each food category (calculated as: % of the difference = (mean amount from 24hR-camera—mean amount from WFR)/ mean amount from WFR)*100)

^b^paired t-test

^c^95% linits of agreenment for the difference between 24hR-camera and WFR, in the corresponding units for each food group and percentage in parenthesis, show the range of under and over-estimation for the agreement between both methods

See also Fig. 2

**Fig. 4:**
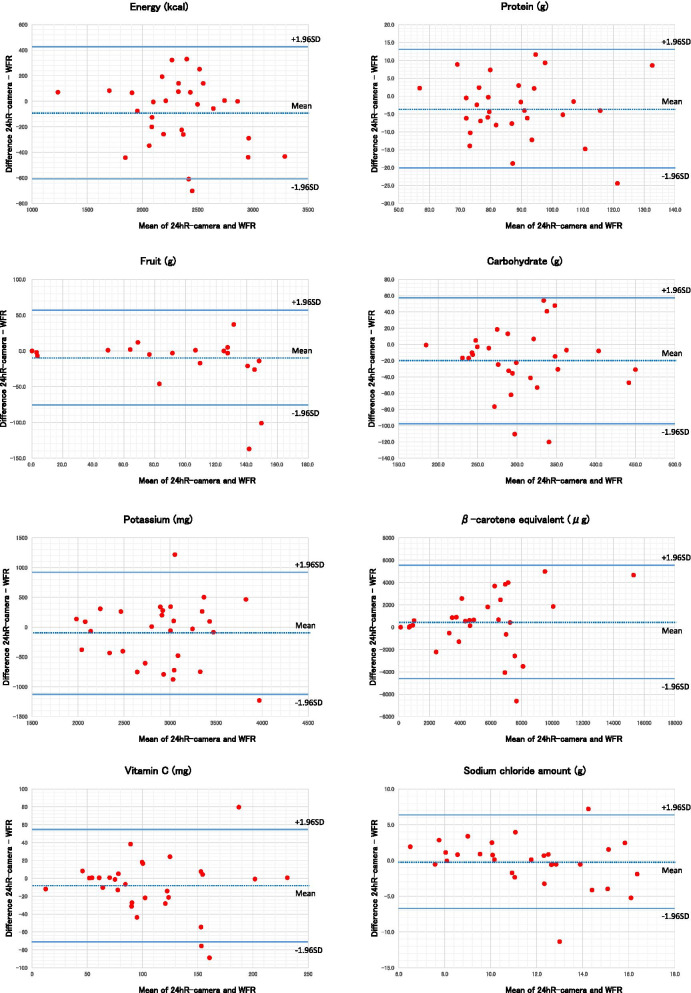
Comparison of intakes of energy and nutrients between 24hR-camera and WFR using Bland–Altman plots. The differences between the amounts of energy and nutrients estimated by 24hR-camera and WFR (Y-axis) were plotted against their mean values (X-axis). The dotted line shows the average difference between the two methods; the smaller the difference, the smaller the systematic bias. The solid lines show the 95% limits of agreement representing the range in which most differences are expected to fall; the smaller the range, the smaller the difference between two methods for most individuals

The estimated vs. weighed nutrient intake is shown in Table 3 and Fig. 4. The correlation coefficients between the estimated vs. weighed were 0.774 for energy and 0.855, 0.769, and 0.763 for the macronutrients: proteins, lipids, and carbohydrates, respectively, showing high correlations with coefficients greater than 0.75. The differences in estimated vs. weighed intake were not significant for energy and lipids, whereas the intake of proteins and carbohydrates were significantly less in the estimated method. The correlation coefficients were 0.583 and 0.560 for salt and potassium intake, respectively, but no significant differences in intake were observed. The correlation coefficients for almost all other nutrients were 0.70 or higher, demonstrating a high correlation.

**Table 3:** Mean difference and correlation coefficient of intakes of energy and nutrients between 24hR-camera and WFR in 30 Japanese men

		24hR-camera	WFR	Difference: 24hR-camera - WFR	P^b^	Correlation coefficient	95% Limits of agreement^c^	
Nutrition		Mean ± SD	Mean ± SD	Mean difference (%)^a^		Spearman (95%CI)		
Energy	kcal	2284.5 ± 401.9	2375.7 ± 457.5	-3.8	0.068	0.774 (0.574, 0.887)	-608.8 (-26.1)	426.3 (18.3)
Protein	g	86.6 ± 16.9	90.1 ± 17.8	-3.8	0.033	0.855 (0.715, 0.929)	-20.0 (-22.7)	13.1 (14.8)
Lipid	g	68.2 ± 23.9	68.2 ± 23.4	0.1	0.985	0.769 (0.565, 0.884)	-27.1 (-39.8)	27.2 (39.9)
Carbohydrate	g	295.6 ± 62.2	315.5 ± 65.2	-6.3	0.010	0.763 (0.555, 0.881)	-97.2 (-31.8)	57.5 (18.8)
Ash	g	20.1 ± 3.3	20.8 ± 4.9	-3.0	0.373	0.668 (0.405, 0.829)	-8.1 (-39.6)	6.8 (33.5)
Potassium	mg	2844.3 ± 559.8	2944.7 ± 584.2	-3.4	0.302	0.560 (0.250, 0.766)	-1125.7 (-38.9)	924.9 (32.0)
Calcium	mg	548.4 ± 227.8	570.7 ± 177.9	-3.9	0.331	0.909 (0.816, 0.956)	-263.6 (-47.1)	219.1 (39.2)
Magnesium	mg	312.2 ± 62.1	326.3 ± 73.9	-4.3	0.158	0.803 (0.623, 0.902)	-118.5 (-37.1)	90.3 (28.3)
Phosphorus	mg	1274.5 ± 255.7	1319.5 ± 256.1	-3.4	0.101	0.838 (0.684, 0.920)	-330.2 (-25.5)	240.2 (18.5)
Iron	mg	8.9 ± 2.0	9.2 ± 2.2	-3.5	0.150	0.908 (0.814, 0.956)	-2.7 (-29.7)	2.0 (22.5)
Zinc	mg	9.6 ± 2.2	10.3 ± 2.4	-6.2	0.001	0.894 (0.787, 0.949)	-2.5 (-25.3)	1.2 (12.5)
Copper	mg	1.4 ± 0.3	1.5 ± 0.3	-7.1	0.006	0.736 (0.511, 0.867)	-0.5 (-34.0)	0.3 (19.3)
Manganese	mg	4.2 ± 2.0	4.2 ± 1.2	0.6	0.931	0.781 (0.585, 0.891)	-3.3 (-77.5)	3.3 (78.8)
Retinol	μg	152.6 ± 83.0	164.4 ± 87.7	-7.1	0.145	0.867 (0.737, 0.935)	-95.9 (-60.5)	72.4 (45.7)
beta-Carotene	μg	5624.0 ± 3782.2	5154.5 ± 3214.8	9.1	0.328	0.735 (0.510, 0.866)	-4596.9 (-85.3)	5536.0 (102.7)
Retinol activity equivalents	μg	625.6 ± 317.8	598.2 ± 264.0	4.6	0.477	0.822 (0.656, 0.912)	-382.1 (-62.4)	437.0 (71.4)
Vitamin D	μg	11.4 ± 11.6	10.6 ± 9.9	7.6	0.418	0.890 (0.780, 0.947)	-9.8 (-88.6)	11.4 (103.3)
α-Tocopherol	mg	8.4 ± 2.8	8.1 ± 2.3	4.2	0.463	0.599 (0.304, 0.789)	-4.6 (-55.4)	5.2 (63.6)
β-Tocopherol	mg	0.5 ± 0.2	0.5 ± 0.2	12.7	0.048	0.727 (0.497, 0.862)	-0.3 (-49.9)	0.4 (73.8)
γ-Tocopherol	mg	13.5 ± 6.3	11.3 ± 4.6	19.8	0.018	0.449 (0.106, 0.697)	-7.3 (-59.2)	11.8 (95.2)
δ-Tocopherol	mg	3.4 ± 1.6	2.7 ± 1.4	23.1	0.003	0.706 (0.464, 0.850)	-1.5 (-49.1)	2.8 (90.6)
Vitamin K	μg	372.7 ± 193.3	390.9 ± 199.5	-4.7	0.359	0.920 (0.837, 0.962)	-228.5 (-59.8)	192.0 (50.3)
Vitamin B_1_	mg	1.2 ± 0.5	1.3 ± 0.5	-5.3	0.177	0.912 (0.822, 0.958)	-0.6 (-47.9)	0.5 (37.0)
Vitamin B_2_	mg	1.4 ± 0.4	1.4 ± 0.4	-4.0	0.078	0.940 (0.877, 0.971)	-0.4 (-28.0)	0.3 (19.8)
Niacin	mg	22.0 ± 7.3	22.4 ± 6.9	-1.8	0.653	0.669 (0.407, 0.829)	-9.7 (-43.6)	8.9 (40.1)
Niacin equivalent	mg	38.1 ± 9.7	39.2 ± 9.4	-2.7	0.327	0.775 (0.575, 0.887)	-12.5 (-32.5)	10.4 (27.0)
Vitamin B_6_	mg	1.6 ± 0.4	1.6 ± 0.5	-2.1	0.508	0.734 (0.508, 0.865)	-0.9 (-56.2)	0.2 (13.0)
Vitamin B_12_	μg	6.6 ± 5.2	6.1 ± 4.2	8.4	0.245	0.903 (0.804, 0.953)	-4.2 (-65.1)	5.2 (81.3)
Folate	μg	377.0 ± 115.1	377.4 ± 84.6	-0.1	0.981	0.797 (0.613, 0.899)	-151.6 (-40.2)	150.9 (40.0)
Pantothenic acid	mg	7.2 ± 1.7	7.6 ± 1.6	-4.7	0.057	0.820 (0.652, 0.911)	-2.3 (-31.2)	1.6 (21.4)
Vitamin C	mg	103.5 ± 51.5	111.7 ± 52.8	-7.3	0.173	0.883 (0.767, 0.943)	-70.9 (-65.9)	54.6 (50.7)
Fatty acid, saturated	g	18.6 ± 7.5	19.2 ± 8.2	-3.1	0.495	0.851 (0.708, 0.927)	-9.7 (-51.4)	8.5 (45.2)
Fatty acid, monounsaturated	g	25.4 ± 10.7	26.1 ± 11.3	-2.4	0.562	0.805 (0.626, 0.903)	-12.2 (-47.4)	10.9 (42.5)
Fatty acid, polyunsaturated	g	15.7 ± 5.8	14.5 ± 4.3	8.2	0.119	0.640 (0.364, 0.813)	-6.8 (-45.1)	9.2 (60.9)
Cholesterol	mg	397.7 ± 219.2	397.9 ± 198.2	-0.1	0.987	0.909 (0.816, 0.956)	-149.6 (-37.6)	149.1 (37.5)
Dietary fiber, soluble	g	4.1 ± 1.3	4.3 ± 1.3	-5.2	0.249	0.748 (0.531, 0.873)	-2.3 (-53.8)	1.8 (43.2)
Dietary fiber, insoluble	g	13.6 ± 5.6	14.2 ± 4.3	-4.3	0.332	0.797 (0.613, 0.899)	-7.3 (-52.5)	6.1 (43.6)
Dietary fiber, total	g	18.5 ± 6.9	19.0 ± 5.4	-3.1	0.484	0.715 (0.478, 0.855)	-9.5 (-50.5)	8.3 (44.3)
Salt equivalents^d^	g	11.6 ± 2.8	11.7 ± 3.7	-1.5	0.772	0.583 (0.282, 0.780)	-6.7 (-57.8)	6.4 (54.8)

*SD* standard deviation, *CI* confidence interval

^a^Persentage of mean difference between 24hR-camera and WFR (calculated as: % of the difference = ((mean nutrient from 24hR-camera—mean nutrient from WFR)/ mean nutrient from WFR)*100)

^b^paired t-test

^c^95% linits of agreenment for the difference between 24hR-camera and WFR, in the corresponding units for each nutrient and percentage in parenthesis, show the range of under and over-estimation for the agreement between both methods

See also Fig. 3

^d^sodium (mg) = salt equivalents (g) × 1000 ÷ 2.54

Estimated (24hR-camera) vs. weighed (WFR) intakes of food were compared using Bland–Altman plots. Figures 3 and 4 show the estimated vs. weighed (Y-axis) plotted against the mean of estimated vs. weighed (X-axis) values for food groups, energy intakes, and nutrients. The dotted line shows the average difference between estimated vs. weighed; the smaller the difference, the smaller the systematic bias. The solid lines show the 95% limits of agreement that represent the range in which most differences are expected to fall; the smaller the range, the smaller the difference between the estimate vs. weighed for most individuals. Extreme outliers, which exceeded the 95% limits of agreement, were rarely seen. The difference between estimated vs. weighed intakes did not correlate with the average of the estimated vs. weighed.

## Discussion

The 24hR-camera method, which combines meal photographs, manual use [2], and interviews with participants, was found to be valid in estimating the intake of foodstuffs and nutrients at a level similar to that of the WFR method, with some exceptions, such as intakes of salt equivalents and vegetables. The correlation coefficient between the estimated (24hR-camera) and weighed (WFR) for salt equivalents intake was low because inconspicuous ingredients, such as oils, fats, condiments, and spices, are difficult to estimate. The intake of vegetables was underestimated in the 24hR-camera method compared to the weight (WFR) method because cooking vegetables reduces their volume, and vegetables are frequently consumed cooked rather than raw in Japan.

While many studies have compared the 24-h dietary recall method and WFR method [5–7], there are only a few studies that have employed camera use to supplement the 24-h dietary recall methods [5, 8, 9]. In a survey of 45 women aged 20 to 52 years in a rural area of Bolivia, the medians of almost all food groups, except drinks and vegetables, were smaller in the 24-h dietary recall with camera method compared to the WFR method. The intake values were lower for almost all nutrients in the 24-h dietary recall method with a camera when compared with the WFR method; however, the Pearson correlation coefficients of nutrition intakes between the methods were high (0.96 or higher) [8]. A 2-day study conducted in England on 10 subjects compared 24-h dietary recall with an automatic camera that took photographs in response to any movement, heat, and light against 24-h dietary recall without a camera. The results demonstrated that “healthy” foods were not over-reported, and “unhealthy” foods were not consistently under-reported [9]. Another study showed the effectiveness of dietary assessment using a camera-equipped portable digital assistant by comparing it with a weighed diet record method. The study subjects were 75 Japanese volunteers, consisting of 27 men and 48 women. The study clearly showed that the method was a useful new dietary assessment method [10], although it is not yet widely employed in the clinical setting.

Our study demonstrated that the correlation coefficients between the estimated (24hR-camera) and weighed (WFR) intakes were at least 0.80 for energy and macronutrients (proteins, lipids, and carbohydrates). We also demonstrated correlation coefficients of 0.7 or higher in almost all food groups, except fats, oils, condiments, and spices (which were 0.3 to 0.5). The correlation was low for salt equivalents, and this is suggested to be due to these ingredients not being visible and the need to be evaluated by taste.

Lazarte et al. [8], reported high correlation coefficients for energy and macronutrients (0.96 or higher, and at least 0.97 for other nutrients). The difference in correlation coefficients with our study may likely be attributed to differences in dietary diversity. While Lazarte et al. recognized the undiversified dietary pattern among their study participants, which likely led to the exceedingly high correlation coefficients, most dishes in Japan contain various ingredients, as exemplified by stew, stir-fried meat, and vegetables, making it difficult for a participant to estimate their food intake. Despite the difficulties in surveying a complex diet, the correlation coefficients of 0.8 or higher for energy and macronutrients imply a sufficiently high correlation, and suggests that the 24hR-camera method is useful for dietary assessment. In a previous study of Japanese people [10], the correlation coefficients between values obtained by a camera-equipped portable device and weighed diet record were lower than those obtained in our study (0.615 for energy, and 0.658, 0.773, 0.708, and 0.408 for protein, fat, carbohydrate, and salt equivalents intake, respectively, in male participants). The authors suggested that the lack of a unified way to take photographs and low quality of digital photographs might have caused the low correlation coefficients. Our study improved on this point using the food atlas, which may explain our higher correlation rate.

The potassium intake calculated by the 24hR-camera and the WFR was different in each food group in terms of absolute value, so the correlation coefficient was low. Especially, intake of vegetables was calculated to be smaller in the 24hR-camrea than in the WFR. Since vegetables become less voluminous when cooked, it seems that the 24hR-camerra underestimated the amount estimation and that led to an underestimation of potassium.

We also compared the 24hR-camera vs. the WFR intake of macronutrients in absolute values. The 24hR-camera intakes of protein and carbohydrate were lower than the WFR. Fish, shellfish, and meat groups shrink when cooked, which could lead to a lower weight intake estimate, resulting in a lower estimate in the protein intake. The reduced carbohydrate intake estimate may be due to a low estimate of the amount of rice consumed. The estimated intakes of micronutrients, such as zinc and copper, were also lower than weighed values; this may be due to the fact that the absolute intake amounts of these nutrients were small.

Our study was novel because only male participants who rarely cooked took part. People who do not cook are likely to have difficulty evaluating the type and amount of foodstuff; therefore, the high correlation in our study, even with male participants who lacked cooking experience, indicated a significant accuracy in the method. It also implies that this dietary assessment method is applicable to other populations, such as women and men, including those who cook on a regular basis.

Taking photographs of actual meals before and after eating is likely to help individuals estimate the correct intake (including leftovers) and prevent them from forgetting to record what they ate or drank [11–19]. There are several advantages to using a camera: 1) easy to distinguish between breakfast, lunch, dinner, snack, and late-night snack; 2) the names of dishes can be objectively determined by an investigator; 3) the food components that make up a dish can be objectively determined by an investigator; 4) the food can be more accurately classified into the food listed in the standard tables of food composition; 5) the type and usage of seasonings and condiments can be confirmed; and 6) weight or wet weight of foods can be more accurately estimated when compared with the 24-h dietary recall method. Therefore, the 24hR-camera method is expected to estimate the intakes of food, energy, and nutrients more accurately than the 24-h dietary recall method, thus enabling a more accurate assessment of nutrient intake. With the increased use of mobile phones, it is now easy for people to take photographs with their mobile camera, making the 24hR-camera method more implementable.

Previous studies validated a method that combined the 24-h dietary recall (without a camera) with the food atlas against the WFR method [20–22]. These studies confirmed that the combination method was useful for participants. Similar to these findings, we were able to obtain high correlations in food groups and nutrients because we used the manual during interviews and implemented a method of taking photographs before and after each meal.

One limitation of our study was that it had a relatively small sample size; however, the number of patients (n = 30) had a statistical power of 80% with a significance level of 5% to detect a true correlation coefficient of 0.5. Most of the correlation coefficients in this study were larger than 0.5. The correlation coefficients for salt equivalents and potassium, which are crucial in the prevention and treatment of lifestyle-related diseases, such as stroke and coronary heart diseases, were smaller than those for other nutrients. We did not confirm the reproducibility of the test methods in this study, and it is the subject of future studies.

One strength of this study was that we developed a dietary assessment method to evaluate male participants who eat Japanese-style meals that commonly have diverse foodstuffs cooked in diverse ways (boiled, stir-fried, deep-fried, etc.), but who also regularly eat Western-style foods such as hamburger steak and stew, as well as Chinese, Spanish, and French cuisines. There is a large variety of food and food preparation in Japan, and thus it is difficult to accurately assess meal intake. The methodology used in this study imposed a relatively small burden on participants, as evidenced by 100% participation in the survey. Finally, because the results from this study pertain to meals consumed under regular life conditions, the method is likely to be feasible in assessing the daily diet, and the results would be internationally comparable.

## Conclusion

The 24-h recall using a portable camera method (24hR-camera) satisfactorily estimated the intakes of energy and macronutrients but not salt equivalents and potassium in Japanese males and was a useful method for dietary assessment.

## Data Availability

The datasets generated and/or analyzed within this study are available from the corresponding author upon reasonable request.

## Abbreviations

WFR: Weighed food records
24hR-camera: 24-Hour dietary recall with the use of a portable camera
